# Cardiac Rhabdomyoma in Adult

**DOI:** 10.7759/cureus.14565

**Published:** 2021-04-19

**Authors:** Somshukla Ghosh, Mark R Milunski

**Affiliations:** 1 Internal Medicine, University of Central Florida College of Medicine, Orlando, USA; 2 Cardiology, Orlando Veterans Affairs Medical Center, Orlando, USA

**Keywords:** tuberous sclerosis complex, benign cardiac tumor, cardiac tumor in adults, surveillance for cardiac rhabdomyoma, tsc-associated cardiac rhabdomyoma

## Abstract

Cardiac rhabdomyoma is a hamartoma comprised of cardiac myocytes. It is the classic cardiac manifestation of tuberous sclerosis complex (TSC) which is an autosomal dominant genetic syndrome with multi-organ involvement, but highly variable phenotype. Cardiac rhabdomyoma is most commonly diagnosed in infancy, 70 to 90% of whom have TSC. However, TSC-associated cardiac rhabdomyoma usually shows spontaneous regression within the first two years of life and hence is extremely rare in adults. We present a 34-year-old woman with TSC who was found to have a cardiac rhabdomyoma when she was referred to the cardiology clinic for evaluation and to establish care. Cardiac rhabdomyoma is usually asymptomatic. However, depending on size and location, it can cause outflow or inflow tract obstruction and aberrant electrical conduction. Hence, appropriate surveillance is important.

## Introduction

Tuberous sclerosis complex (TSC) is an autosomal dominant genetic syndrome associated with multi-organ involvement including cardiac rhabdomyomas. Cardiac rhabdomyoma is a benign tumor comprised of cardiac myocytes. They are most commonly found in the ventricles, but rarely can be located in the atria. Echocardiography is the imaging modality of choice for screening in patients with TSC. Cardiac rhabdomyoma is usually asymptomatic. However, they can interfere with valvular function, cause outflow or inflow tract obstruction and aberrant electrical conduction leading to heart failure and lethal arrhythmias [1].

## Case presentation

A 34-year-old woman with known TSC (as per clinical diagnostic criteria [2]) was referred to the cardiology clinic for further evaluation and to establish care. The patient had multisystem involvement with numerous angiofibromas on the face, a subependymal nodule at the level of the left foramen of Monroe and lymphangioleiomyomatosis of lungs. She was noted to have abnormal skin lesions since she was 13 years of age, however diagnosis of TSC was not suspected until years later when she consulted a dermatologist for her skin lesions.

**Figure 1:**
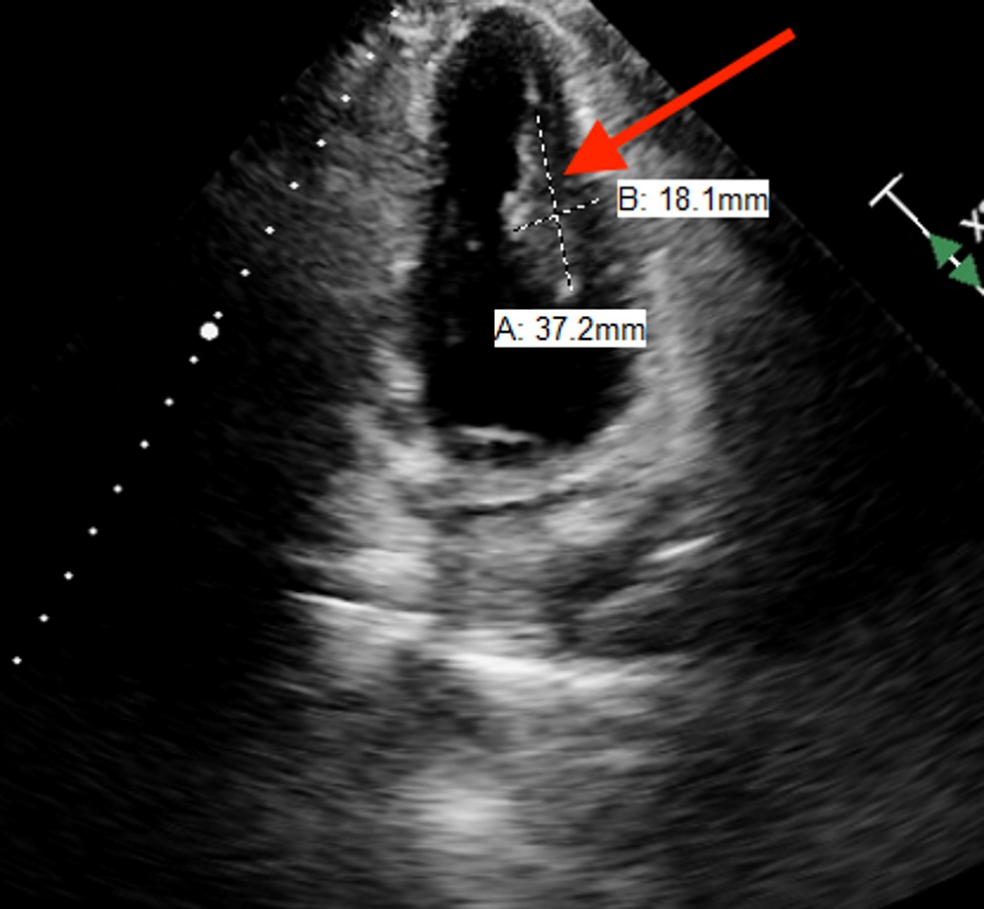
Rhabdomyoma arising from lateral wall of left ventricle and protruding into the left ventricular cavity

**Figure 2:**
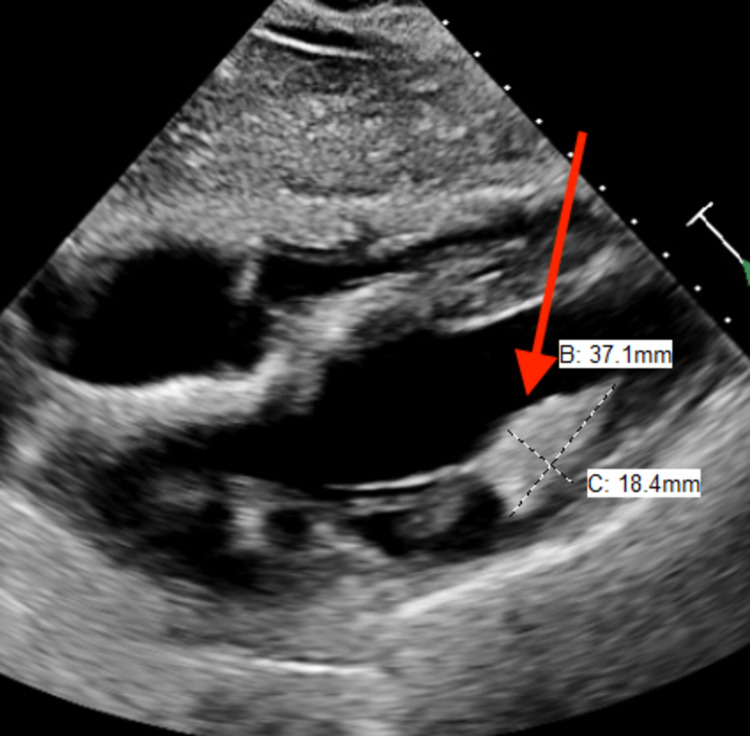
Parasternal long axis view showing rhabdomyoma arising from lateral wall of left ventricle and protruding into the left ventricular cavity

She denied chest pain, dyspnea, palpitations, peripheral edema, or syncope. Vital signs were normal. Physical examination revealed a normal S1 and S2 with regular rhythm and a rate of 84/min. There was no murmur. Lungs were clear to auscultation bilaterally with no adventitious sounds. Dermatological exam revealed multiple angiofibromas on the face and fingers. Electrocardiogram (ECG) revealed normal sinus rhythm. Echocardiography revealed a well-circumscribed 37mm x 18mm echo-dense, homogeneous mass attached to the lateral wall of left ventricle (Figures 1, 2). It was hyperechoic compared with the adjacent myocardium, consistent with rhabdomyoma. Left ventricular systolic function was normal. Doppler echocardiography did not show any left ventricular inflow or outflow tract obstruction. Both the atria were normal in size. The right ventricle was normal in size and function. There was no stenosis or significant regurgitation across any of the valves. A 14-day ambulatory event monitor did not reveal any significant arrhythmias. She was recommended surveillance with yearly ECG and echocardiogram. Since then she has continued to be asymptomatic and annual ECG and echocardiogram for five consecutive years have not shown any significant changes.

## Discussion

Cardiac rhabdomyoma is usually diagnosed in infancy, with 70% to 90% of these children having TSC [3-5]. However, TSC-associated cardiac rhabdomyomas commonly regress spontaneously within the first two years of life, thus being extremely rare in adults. They are hamartomatous growths and literature review does not reveal any evidence for a potential malignant transformation. Most commonly, cardiac rhabdomyomas are present in multiple sites in the myocardium and individual rhabdomyomas may vary in size from a few millimeters to several centimeters. Typically they are located in the ventricles with equal possibility of arising from the left, right or septal ventricular myocardium [6-7]. Rarely they can be located in the atria. Patients with cardiac rhabdomyomas are generally asymptomatic. However, depending on size and location, these tumors can interfere with valvular function, cause inflow or outflow tract obstruction or aberrant electrical conduction leading to heart failure or lethal arrhythmias [8-9]. Also, if located in the atria they can result in compromise of the coronary circulation by compressing the coronary arteries and hence cause myocardial ischemia [10].

Thus, all patients with TSC should be screened for cardiac rhabdomyomas. Echocardiography with complete Doppler study is the imaging modality of choice for patients with TSC. Typically, on echocardiogram they are seen as well-circumscribed, echogenic, multiple nodular masses within the myocardium, occasionally projecting into the corresponding cardiac chamber and are homogeneous and hyperechoic compared to the surrounding myocardium. Diagnosis of cardiac rhabdomyomas is easy in the presence of these typical features. However, diagnosis can be difficult if they are present in an atypical location like the atria or occur as a solitary tumor. In that case the echocardiogram can be followed up with a cardiac MRI which helps with better tissue delineation. An endomyocardial biopsy is rarely required for diagnosis of a cardiac rhabdomyoma, particularly in a patient like ours, with already diagnosed TSC, given such a high association of TSC with cardiac rhabdomyoma.

The American Heart Association recommends surveillance with an ECG every three to five years in asymptomatic adults to monitor for conduction defects or abnormal rhythms [1]. However, our patient was recommended surveillance with yearly ECG and echocardiography due to the large size of the tumor.

## Conclusions

Cardiac rhabdomyoma is extremely rare in adults. However, adults diagnosed with TSC should undergo appropriate cardiac imaging and surveillance because of the risk of heart failure and arrhythmias associated with these tumors.
